# The Dose- and Time-Dependent Cytotoxic Effect of Graphene Nanoplatelets: In Vitro and In Vivo Study

**DOI:** 10.3390/nano12121978

**Published:** 2022-06-09

**Authors:** Hana Bavorova, Tereza Svadlakova, Zdenek Fiala, Rishikaysh Pisal, Jaroslav Mokry

**Affiliations:** 1 Department of Histology and Embryology, Faculty of Medicine in Hradec Kralove, Charles University, 50003 Hradec Kralove, Czech Republic; pisalr@lfhk.cuni.cz (R.P.); mokry@lfhk.cuni.cz (J.M.); 2 Institute of Clinical Immunology and Allergology, University Hospital Hradec Kralove and Faculty of Medicine in Hradec Kralove, Charles University, 50005 Hradec Kralove, Czech Republic; svadlakovat@lfhk.cuni.cz; 3 Institute of Preventive Medicine, Faculty of Medicine in Hradec Kralove, Charles University, 50003 Hradec Kralove, Czech Republic; fiala@lfhk.cuni.cz

**Keywords:** graphene nanoplatelets, cytotoxicity, nanomaterials, PAECs, C57Bl/6 mice

## Abstract

Graphene-based nanomaterials received attention from scientists due to their unique properties: they are highly conductive, mechanically resistant and elastic. These materials can be used in different sectors of society from electronic energy storage in industry to biomedical applications. This study evaluates the influence of graphene nanoplatelets in vitro and in vivo. The toxicological influence of graphene nanoplatelets (GPs) was analyzed by cytotoxic methods, the change of cell proliferation was assessed in real-time, and the effect of GPs on a living organism was evaluated in an animal model using histopathological examination. We analyzed two types of GP administration: intratracheal and peroral. We found dose- and time-dependent cytotoxic effects of GPs in vitro; the concentration above 50 μg/mL increased the cytotoxicity significantly. The real-time analysis confirmed these data; the cells exposed to a high concentration of GPs for a longer time period resulted in a decrease in cell index which indicated lower cell viability. Histopathological examination revealed thickened alveolar septa and accumulation of GPs in the endocardium after intratracheal exposure. Peroral administration did not reveal any morphological changes. This study showed the dose- and time-dependent cytotoxic potential of graphene nanoplatelets in in vitro and in vivo models.

## 1. Introduction

Nanomaterials are becoming increasingly common in industry. Their widespread utilization also considerably increased interest in medical research and supports the need for a comprehensive assessment of their potential impact on human health. Industry [1,2], the biomedical field (drug or gene delivery, bioimaging [3,4]) and many more sectors use nanomaterials of various shapes, types and amounts. The “miracle material”, graphene, is a 2D nanomaterial that provides a broad spectrum of properties including mechanical stiffness, strength, elasticity, electrical and thermal conductivity and many others [5]. Graphene is a carbonaceous nanomaterial derived from crystalline graphite [6] that has a honeycomb-like network shape [7], but there is a wide range of methods for fabricating these graphene-based nanomaterials (GBNMs) leading to the production of different shapes, including nano-sheets, nano-platelets, nano-ribbons, nano-quantum dots, nano-shells and many more [8,9]. Graphene nanoplatelets (GPs) can be utilized in many applications such as oxygen evolution reactions, photocatalysis, electrochemical sensors and amperometric biosensors [10,11,12,13,14,15].

The biohazardous potential of GBNMs has been a subject of intense research in the scientific community. However, graphene-based nanomaterials are produced in many forms and differences in their fabrication and post-processing have a significant impact on their properties and can potentially increase toxicity to living organisms [16,17,18]. This is the main reason for the growing number of peer-reviewed articles confirming the need for comprehensive studies to understand the toxicity potential of GBNMs. The results of studies investigating the toxicity and biocompatibility of GBNMs (Table S1) vary widely, with studies reporting lower or no toxicological effect on cell lineages exposed to the GPs [19], while other studies confirm time- and dose-dependent toxicity [20,21].

The ultimate goal is to properly assess the toxicity to the living organisms (Table S2). The biological distribution of graphene-based nanomaterials has been a subject of research for as long as nanomaterials have been fabricated. Yet, there are no comprehensive data describing the exact process of its fate in the body. To better understand the fate of GBNMs, we must first know the complete nature of the nanomaterial, which can lead to presumptions about its behavior in the body. We must consider the internal environment of the living organism because it can dramatically affect biological behavior [22]. For example, Kurapati et al. studied the biodegradation of GBNMs and focused on two different types—single layer and multilayer graphene. They proved that graphene flakes can be processed and destroyed by neutrophils [23]. These findings could have an important clinical impact and suggest the usage of those materials in the human body where is a presumption of complete biodegradation.

Our study describes the in vitro and in vivo effect of a well-characterized graphene-based nanomaterial in the form of a platelet with a size of up to 2 μm and a thickness of 1–4 nm. For in vitro study, we used cells isolated from the lung tissue to examine the potential deteriorative impact on cell proliferation. The cytotoxicity level was minimal in low doses, but high concentrations (50–100 μg/mL) reduced cell proliferation and survival. C57Bl/6 mice were used to study the in vivo toxicity of graphene nanoplatelets and we used two different approaches to deliver the nanomaterial in the body (the intratracheal and peroral pathway). The histopathological examinations of major organs impacted by exposition to the nanomaterial were performed to explore potential morphological alterations and the fate of nanomaterials in living tissues.

## 2. Materials and Methods

### 2.1. Fabrication of the Graphene Nanomaterial

In our study, we used graphene platelets (GPs) obtained from PlasmaChem GmbH in the form of a powder (product number PL-P-G750, Berlin, Germany). According to the manufacturer’s specifications, the particle size was up to 2 μm and the thickness of the graphene sheets was 1–4 nm. The nanomaterial was thoroughly investigated for composition, structure and thermal stability. The results of the X-ray diffractometry, energy dispersive X-rays spectroscopy, Raman spectroscopy and thermogravimetric analyses have been recently published by Svadlakova et al. [24]. Briefly, all obtained physicochemical characterization results were in line with the specifications of the producers of these materials.

### 2.2. Preparation of Suspensions

Stock suspensions of GPs at a concentration of 250 μg/mL were prepared by dispersing powder in 0.02% sodium cholate and sonicated by a sonic probe (QSonica, Q700 ultrasonic processor, LLC, Newtown, CT, USA) for 30 min with a 65% amplitude. The hydrodynamic diameter and zeta potential of GPs in suspension further diluted in sterile water or culture medium (10% FBS) were determined using Zetasizer Nano-Ultra (Malvern Panalytical Ltd., Malvern, UK). The absence of biological contamination was confirmed using a cell-based assay as previously stated by Svadlakova et al. [25]. The stock solution was diluted in different concentrations and exposed to cell culture media with 10% FBS.

### 2.3. Mouse Primary Alveolar Epithelial Cells Culture (PAECs)

C57BL/6 mouse primary alveolar epithelial cells were isolated from lung tissue of pathogen-free laboratory mice (Cell Biologics). Cells were cultured in an epithelial cell medium (Cell Biologics) with growth factors (0.01% insulin-transferrin-selenium, 0.01% EGF, 1% L-glutamine, 1% antibiotic-antimycotic solution, 10% FBS, Cell Biologics, Chicago, IL, USA). Cells were cultured in T75 flasks (VWR) in a 5% CO_2_ humidified incubator (Thermo Fisher Scientific, Waltham, MA, USA) at 37 °C.

### 2.4. Cytotoxicity Assessment

Cell proliferation and viability were determined with several cytotoxic methods studying the influence of GPs co-cultured with PAECs for 24 and 48 h. The CyQUANT LDH cytotoxicity assay (ThermoFisher Scientific, Waltham, MA, USA) was one of the methods used to measure the cytotoxicity effect on cells. Briefly, PAECs in different densities (0.5–1 × 10^4^ cells) were incubated overnight in a 96-well plate, and cells were washed and exposed to increasing concentrations of GPs (5–100 μg/mL) for 24 or 48 h. The lactate dehydrogenase (LDH) assay was performed according to the manufacturer’s protocol. Absorbance was measured in a SUNRISE Xfluor4 (TECAN, Männedorf, Switzerland) microplate spectrophotometer at 492 nm. All experiments were performed in triplicates for each sample.

To determine the effect of graphene on the proliferation of PAECs, a WST-1 assay (Sigma, Buenos Aires, Argentina) was used. Cells were added to a 96-well plate and cultured for 24 h. For another 24 to 48 h, the GPs were added to the culture. Then, tetrazolium salt (WST-1) was pipetted and incubated with exposed cells for 4 h. Finally, the colored product was analyzed with an ELISA analyzer (TECAN) at 490 nm. All experiments were performed in triplicate for each set of samples.

### 2.5. Real-Time Cell Analysis

The DP version of the xCELLigence system (Agilent Technologies, Inc., Santa Clara, CA, USA) was used throughout these analyses, which comprised 3 measuring stations (each consisting of 16 wells) controlled independently. The xCELLigence DP system measured the electrical impedance in the bottom of the well and converted it to the Cell Index.

An optimal seeding density for PAECs was 1000 cells per well. Dynamic cell proliferation was monitored in 15 min intervals from the plating time until exposure to the nanomaterial (24 h). To determine the sensitivity of PAECs to the nanomaterial, the graphene was added to each well in different concentrations (5–100 µg/mL) for 24 or 48 h except for the cell used as a control. Data were processed and plotted using the xCELLigence software package.

### 2.6. In Vivo Study

Sixty-six adult male C57Bl/6 mice (8–12 weeks age, body weight of approx. 25 g) were housed in polyethylene cages and maintained under a controlled temperature and a 12 h light/12 h dark cycle for 1 week before the start of the experiment. They were allowed ad libitum access to standard pellets and tap water. Animals received humane care; all the experiments were performed in accordance with the international guidelines and were approved by the Ethical Committee of the Ministry of Education, Youth and Sports of Czech Republic (approval No. MSMT-9237/2020-2).

Mice were randomly divided into eight groups of 6 to 9 animals per group (Table 1). Group 1 received GPs intratracheally (IT) in two different concentrations (5 or 50 ug/mL), and group 1C was exposed on working days for 21 days (chronic exposure). The rest of group 1 received only one dose of GPs (acute exposure). Group 1D was assessed as a vehicle control group, where sodium cholate was administered (0.02%). Group 2 was exposed to the GPs perorally (PO) with different concentrations of GPs (5 or 50 μg/mL), and group 2C was assessed as a chronic exposure group with the same conditions as group 1C. Group 2D, which was exposed to sodium cholate only, was considered a control group. After 1, 7 or 21 days after the last dose, the animals were euthanized and organs were harvested for further histological analysis.

Removed organs were fixed in 10% formalin, embedded in paraffin and sectioned into 5 μm sections. Sections were deparaffinized, rehydrated and washed with distilled water. The sections were stained with hematoxylin and eosin.

### 2.7. Statistical Analysis

Unless stated otherwise, the data are shown as mean values (*n*-tests = 3) ± standard deviation and are normalized to the control. Changes are considered significant for *p*-values < 0.05. Based on the Shapiro–Wilk test of normality, either the parametric or nonparametric analysis of variance (ANOVA) followed by Dunnett’s test was performed using GraphPad Prism^TM^ software, version 9.3.1 (GraphPad Software Inc., San Diego, CA, USA).

## 3. Results

### 3.1. Characterization of the Graphene Nanoplatelets

The characterization of graphene nanoplatelets is summarized and published in the article by Svadlakova et al. [24].

### 3.2. Cytotoxicity Analysis and Cell Morphology Evaluation

**Figure 1:**
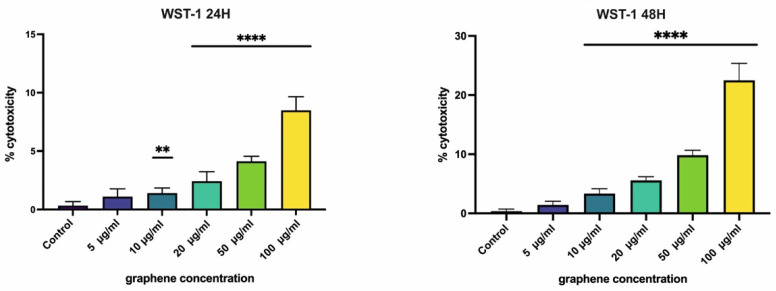
Cytotoxicity of nanomaterials determined by WST-1 analysis. The cytotoxicity level increased with longer exposure to the GPs. Data are presented as a percentage (%) of untreated control (0 μg/mL) and are visualized as mean ± standard deviation. ** *p*-value < 0.01; **** *p*-value < 0.0001.

Cellular survival can be evaluated by tetrazolium-based methods. In the WST-1 assay, the amount of dye generated is directly proportional to the number of living cells. The results indicated that the cells exposed to a high concentration of GPs (50 or 100 µg/mL) had an increasing cytotoxicity percentage (Figure 1). There was also a relation to exposure time, where cells incubated with GPs for 48 h had a moderately higher number of dead cells compared to the group exposed to GPs for 24 h only. However, the percentage did of dead cells not exceed 25% of the total amount of cells.

**Figure 2:**
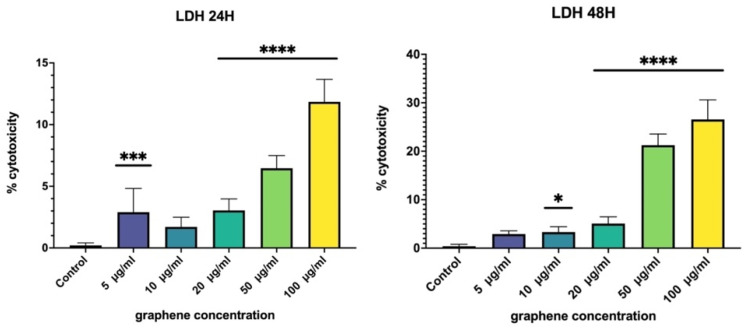
Cytotoxicity of nanomaterials determined by LDH analysis. The cytotoxicity level increased with longer exposure to the GPs. Data are presented as a percentage (%) of untreated control (0 μg/mL) and are visualized as mean ± standard deviation. * *p*-value < 0.05; *** *p*-value < 0.001; **** *p*-value < 0.0001.

Similar results related to the time-dependent cell survival ratio were determined with the LDH cytotoxicity assay. The LDH assay is used to reflect the extent of the plasma membrane damage and the released LDH in the cell culture medium can be quantified; the level of formazan formation is directly proportional to the amount of the damaged cells. Low concentrations of GPs (5–20 µg/mL) did not have any significant impact on the cell survival ratio, even if the time exposure was 48 h (Figure 2). However, the cytotoxicity level increased above 20% after longer exposure (48 h) and with a high concentration of the GPs (time- and dose-dependent cytotoxic effect).

### 3.3. Real–Time Analysis of Cell Growth

The characterization of the potential anti-proliferative effect on PAECs was assessed with real-time analysis of cell growth. The changing cell index (change of electrical impedance represents the cell status) reflects differences in cell morphology, adhesion and/or viability. This value corresponds to the strength of the cell adhesion and cell number (if the cell dies, the cell index value decrease).

**Figure 3:**
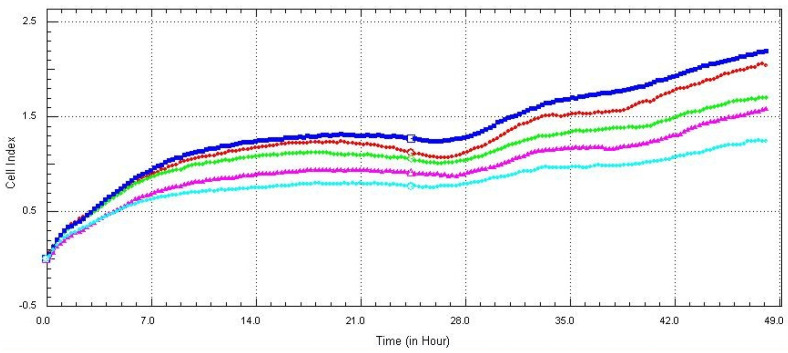
Real–time analysis of (RTCA) screening of PAECs. Cells were seeded at 0 h and GPs were administrated at 24 h. Cell growth was then monitored for the following 24 h. Deep blue (5 µg/mL), deep red (10 µg/mL), green (20 µg/mL), purple (50 µg/mL), turquoise (100 µg/mL).

**Figure 4:**
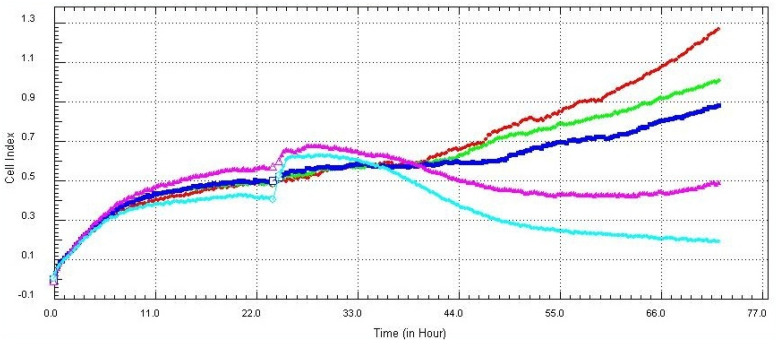
RTCA screening of PAECs. Cells were seeded at 0 h and GPs were administrated at 24 h. Cell growth was monitored for the following 48 h. Deep red (5 µg/mL), green (10 µg/mL), deep blue (20 µg/mL), purple (50 µg/mL), turquoise (100 µg/mL).

The normalized cell index (CI) value of PAECs remained without any significant changes after 24 h of exposition to the GPs; the CI value even constantly increases, suggesting that PAECs maintained the cell monolayer and consolidated the cell–cell attachment (Figure 3). A significant decrease was observed after exposure to a high concentration of GPs (50 and 100 µg/mL) and after prolonged exposure (48 h), and the normalized cell index decreased to 0.5 in the first 9 h after exposure to the GPs. The decline gradually decreased and remain constant until the end of exposure (Figure 4), which indicates that GPs induced oxidation stress and disrupted the cell barrier leading to a discontinuous cell monolayer.

### 3.4. Histopathological Findings

We also analyzed the effects of GPs on a living organism by delivering the nanomaterial to the C57Bl/6 mice in two ways (intratracheal and peroral), which corresponds to possible routes of entry into the human body.

**Figure 5:**
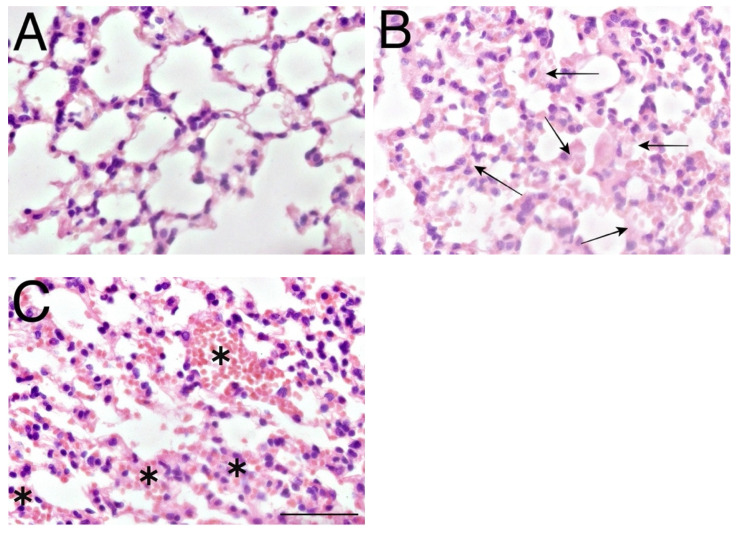
Histopathological effects of GPs on the lung tissue of C57Bl/6 mice after 21 days. Representative microphotograph of control mice group (**A**); (**B**,**C**) mice exposed to 50 µg/mL GPs. Arrows indicate a thickened alveolar wall; asterisks indicate red blood cells in the lung interstitial tissue. The scale bar = 50 µm, staining haematoxylin-eosin.

After the intratracheal exposition, we expected an accumulation of GPs in animals exposed periodically (chronic exposure), but we observed the presence of GPs only in one sample (the heart tissue). The main morphological changes were apparent after 30 days of exposure, periodically repeated every working day to mimic the exposure of workers in daily contact with nanomaterials. Figure 5B,C shows microphotographs of lung tissue exposed to 50 µg/mL of GPs with thickened alveolar walls and abundant erythrocytes flooding the interstitial connective tissue. The thickness of alveolar walls was significantly different from the control group. The quantification revealed that the size of the alveolar septa increased in size three times compared to the control sample (Figure S1). The usual thickness of the alveolar septa of the control group was approx. 3 µm, but chronic exposure caused an increase to 9 µm. However, there was an absence of inflammation in the lung tissue. We did not detect any changes in morphology in the control group (Figure 5A) that received the sodium cholate (used as a solvent for GPs).

**Figure 6:**
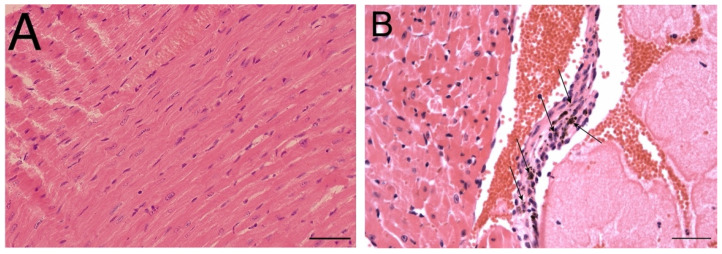
Histopathological effects of GPs on the heart tissue of C57Bl/6 mice after 21 days. Representative photomicrographs of control mice group (**A**) and (**B**) mice exposed 50 µg/mL of GPs. Arrows indicate the accumulation of GPs in the endocardium of the heart. Cardiomyocytes in the control group have physiological morphology. The scale bar (**A**) = 50 µm, (**B**) = 20 µm; staining haematoxylin-eosin.

Heart tissue was also collected after intratracheal exposure. No histopathological lesions were observed in the heart tissue of the control group of mice (Figure 6A). However, chronic exposure revealed tiny specks of GPs (Figure 6B) in the loose connective tissue of the endocardium, but the morphology of the myocardium was not affected. The mice were exposed daily to the GPs chronically with high doses (50 µg/mL); low doses (5–20 µg/mL) of GPs did not lead to any changes in morphology.

**Figure 7:**
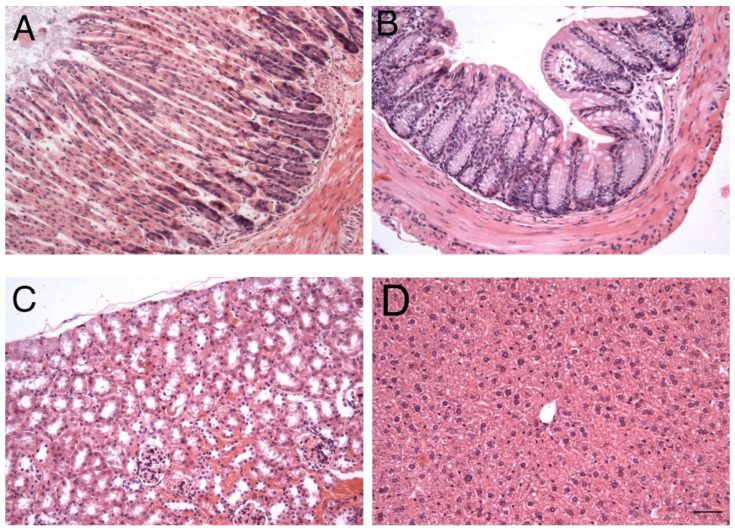
Effects of GPs on the stomach (**A**), small intestine (**B**), kidney (**C**) and liver (**D**) of C57Bl/6 mice after 21 days of oral exposure. The nanomaterial did not change the tissue morphology of listed organs; their microstructure was identical to the control group. The scale bar = 20 µm; staining haematoxylin-eosin.

Histopathological changes in organs involved in the oral exposure to the GPs were not significant enough, even for the group with chronic exposure. We examined the stomach (Figure 7A), small intestine (Figure 7B), kidneys (Figure 7C) and liver (Figure 7D) to compare their morphology to the control group. Both groups (acute and chronic exposure) were comparable to the control group, the histological analysis showed no changes in the tissue of the organs examined. Some studies described deposits of graphene in the parenchyma [26,27] or in the capsule, but these were absent in our samples. We were not able to observe any GPs in cells even in samples after chronic exposure to GPs. That was most likely caused by low GPs concentration and also due to the animal’s capacity to metabolize or excrete the nanomaterial from their bodies. Oral exposure to the GPs was in no way harmful to small animals, since any inflammation, accumulation of nanomaterials or giant cell formation was not evident in the samples. We conclude that intratracheal exposure has more detrimental effects than oral GP administration.

## 4. Discussion

Studies concerning the safety of nanomaterials for living organisms are lacking, mainly due to the enormous amount of newly fabricated types of nanomaterials. The variation in quality, shape or oxidation of nanomaterials results in a wide diversity of their toxicological effects on living organisms [28]. The aim of our study was to contribute to the knowledge of nanomaterials’ behavior through in vitro and in vivo environments.

The main aim of this study was to evaluate the effect of GPs on normal healthy cells as well as on living organisms. Our data prove the dose- and time-dependent cytotoxic influence of GPs on cell lineages. These conclusions were also observed by other studies focused on the cytotoxic properties of GPs [20,29]. A possible toxicological mechanism behind these findings is the physical properties of GPs and due to the sharp edges of graphene mechanically distorting the plasmatic membrane of the cells [30]. The ratio of living cells was assessed with WST-1, which showed better results by investigating the viability than commonly used MTT methods which usually overestimate cytotoxicity levels [31]. The enzymatic reactions involved in the WST-1 assays seem to not be affected by the presence of graphene nanomaterials. In the present study, the toxicity effect was significant in the cell culture exposed to high concentrations (50–100 µg/mL) of GPs, but the decrease in viability did not exceed 25% of the total amount (Figure 1 and Figure 2). Similar results were obtained in the study concerning the toxicity of graphene oxide to A549 cells [32] comparing different types of graphene-based nanomaterials to determine their biocompatibility. Wang et al. [27] suggested that the level of biocompatibility correlated with the dose and length of exposure.

The membrane integrity could be assessed by morphological methods such as transmission electron microscopy or with biochemical approaches such as the LDH assay used in our study. When the cytoplasmic membrane is damaged, the intracellular LDH is released into the culture medium and could be quantified. Therefore, the LDH level correlates with cell damage and resistance of the cell membrane. The GPs did not induce significant leakage of LDH into the cytoplasm of PAEC’s cell culture 24 h after exposure to high doses (Figure 2). However, after prolonged exposure (48 h) and with high doses (50–100 µg/mL), the release of LDH is doubled compared to after short-term exposure (Figure 2). However, the increase is not significant enough to declare GPs as a highly toxic nanomaterial. Similar data were also published by other studies [33,34].

Monitoring the biological status of cells in real-time after their exposure to nanomaterials is a technique helping to determine changes in cell number, adhesion, viability and morphology. Only a few studies used this method to estimate the influence of nanomaterials on the cell culture [35,36,37]. These results showed the same tendency as was analyzed by the WST-1 test: high concentrations of GPs and prolonged exposure most likely caused disruption of the cell membrane and decreased cell viability. It should be highlighted that we used healthy non-cancerous cells in our experiments, but the majority of studies use cancerous cell lineages, which could cause different behavior and responses to the nanomaterials compared to healthy cell lineages [35,38]. However, our results showed accordance with their findings, which led us to the conclusion that high concentrations of nanomaterials could potentially result in oxidative stress due to their accumulation in the cytoplasm followed by membrane rupture. This phenomenon was successfully adopted by Razaghi et al. [39]. They used fluorinated graphene oxide as an MRI agent, and it also had the capacity to load hydrophobic therapeutic agents. They combined a fluorinated graphene oxide with a linoleic acid-curcumin conjugate (used as an anticancer drug) and it resulted in the accumulation of anticancer agents in the cancer cell.

The small diameter of the GPs makes them easily respirable, which could result in the accumulation of small particles in the lung tissue as described by Gao et al. [40]. They performed nose inhalation in rats and analyzed subsequent aggregation of GPs in the alveolar macrophages. Our data did not confirm these findings which might be due to the different respiratory parameters of mice used in our study. Histopathological analysis revealed thickened alveolar septa, which could be an inflammatory response to daily exposure to GPs in high concentrations (50 µg/mL). Similar findings were confirmed by Shin et al. [41]; they used rats inhaling GPs for 5 days, 6 h/day using an atomizer. They found slight thickening of the alveolar wall and that alveolar macrophages ingested the GPs. Due to the close relationship between the cardiovascular and respiratory systems, we analyzed samples of heart tissue after intratracheal exposure to the animals. We observed an accumulation of small GP particles in the endocardium, but no signs of myocardial hypertrophy or inflammation. Kanakia et al. [42] confirmed histopathological changes in heart of Wistar rats after their exposure to dextran-coated graphene oxide nanoplatelets. Their myocardium contains focal congestion with hyper-eosinophilic cardiomyocytes, and they also measured blood pressure after intravenous injection of graphene, which could not be obtained until 2 h after the injection. These findings were observed with the usage of a much higher concentration of nanomaterials from 250 to 500 mg/kg.

Neither acute nor chronic exposure to GPs by oral administration resulted in histopathological changes in the organs collected for the evaluation. We concluded that mice were able to manage high concentrations of GPs, metabolize them and subsequently excrete them without any damage. Oral administration of graphene oxide was reported by Fu et al. [43] and they used graphene oxide dissolved in water (concentration was 0.05 and 0.5 mg/mL) and then the mixture was served to gravid mice for up to 38 days. Then they examined the filial mice and noticed significant retardation of growth in the group exposed to 0.5 mg/mL of graphene oxide; the maternal mice had no behavioral or weight disorder compared to the control group. The toxicological mechanism of graphene is based on the generation of reactive oxygen species, which could initiate oxidative stress in the cell and is then followed by DNA damage and potentially necrosis or apoptosis [44]. These results showed that the modification of nanomaterials, the used concentration and the frequency of oral exposure could have a significant influence on the toxicity of graphene.

## 5. Conclusions

Our investigation of the cytotoxicity effect of graphene nanoplatelets was focused on commonly used methods to investigate changes in the cell proliferation or integrity of cell membranes. Our findings demonstrated that GPs could induce damage to cell lineage after prolonged exposure and if high concentrations were used. Real-time analysis of cells exposed to nanomaterial also showed that GPs had time- and dose-dependent toxicity due to the decreased cell index, suggesting damage to the cell monolayer and cell membrane. GPs could enter the lung tissue and cause inflammation, resulting in thickened alveolar septa, and could accumulate in the endocardium after intratracheal exposure to the nanomaterial. However, chronic exposure to GPs by oral administration did not show any pathological changes in the morphology of different organs compared to the control group, which led us to the conclusion that the route of exposure had a significant impact on the toxicity of GPs. There is a need for further studies to investigate the exact toxic mechanism of graphene nanoplatelets to the living organism, but our findings might provide material for the biological behavior of GPs in the body.

## Figures and Tables

**Table 1:** Animal groups, doses and application routes of GPs.

Group	Exposure Routes	Dosing Solution	No. of Animals	Exposure
1A	IT	5 μg/mL	9	1, 7, 21 days
1B	IT	50 μg/mL	9	1, 7, 21 days
1C	IT	50 μg/mL	6	21 days
1D	IT	0 μg/mL	9	1, 7, 21 days
2A	PO	5 μg/mL	9	1, 7, 21 days
2B	PO	50 μg/mL	9	1, 7, 21 days
2C	PO	50 μg/mL	6	21 days
2D	PO	0 μg/mL	9	1, 7, 21 days

## Data Availability

The data presented in this study are available on request from the corresponding author.

## Supplementary Materials

The following supporting information can be downloaded at: https://www.mdpi.com/article/10.3390/nano12121978/s1, Figure S1: Quantification of GPs influence to lung tissue after chronic exposure (21 days); Table S1: In vitro toxicity of the graphene-based nanomaterials; Table S2: In vivo toxicity of the graphene-based nanomaterials. References [45,46,47,48,49,50,51] are cited in the Supplementary Materials.
