# Supplementary material for: The Dose- and Time-Dependent Cytotoxic Effect of Graphene Nanoplatelets: In Vitro and In Vivo Study
Source: Nanomaterials (Basel). 2022 Jun 9;12(12):1978. doi: 10.3390/nano12121978 (PMC9229803; doi:10.3390/nano12121978)
Supplement: Supplementary file 1 [file nanomaterials-12-01978-s001.zip › nanomaterials-1747611-Supplemental Materials.pdf]

Supplemental Materials

# The Dose- and Time-Dependent Cytotoxic Effect of Graphene Nanoplatelets: In Vitro and In Vivo Study

**Hana Bavorova [1,*], Tereza Svadlakova [2,3], Zdenek Fiala [3], Rishikaysh Pisal [1] and Jaroslav Mokry [1]**

1 Department of Histology and Embryology, Faculty of Medicine in Hradec Kralove, Charles University, 50003 Hradec Kralove, Czech Republic; pisalr@lfhk.cuni.cz (R.P.); mokry@lfhk.cuni.cz (J.M.)
2 Institute of Clinical Immunology and Allergology, University Hospital Hradec Kralove and Faculty of Medicine in Hradec Kralove, Charles University, 50005 Hradec Kralove, Czech Republic; svadlakovat@lfhk.cuni.cz
3 Institute of Preventive Medicine, Faculty of Medicine in Hradec Kralove, Charles University, 50003 Hradec Kralove, Czech Republic; fiala@lfhk.cuni.cz
* Correspondence: hrebikh@lfhk.cuni.cz or cze-mail@e-mail.com

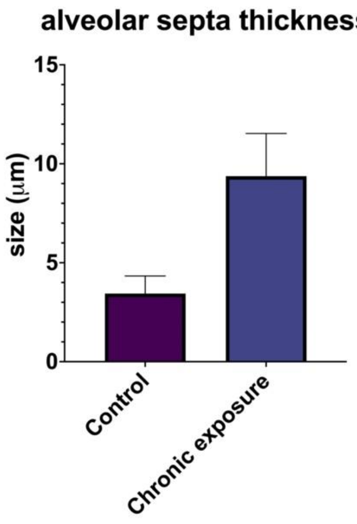


**Figure S1.** Quantification of GPs influence to lung tissue after chronic exposure (21 days).

**Table S1.** Table S1: In vitro toxicity of the graphene-based nanomaterials.

| Tested nanomaterial | Cytotoxic assay | Concentration | Cell culture | Findings | Reference |
|---|---|---|---|---|---|
| graphene oxide (GO), reduced GO,graphene quantum dots (GQD), aminated GQD, carboxyl GQD, graphene oxide GQD | MTT, LDH, TUNEL, ROS, live/dead cell staining | 12.5, 25, 50, 100, 200 µg/ml | HL-7702 cells, SMMC-7721 cells, BEL-7404 cells, HCT116 cells, PC-12 cells, | Dose and time dependent cytotoxicity (for GO, rGO); other particles showed no obvious cytotoxicity | Li et al. (1) |
| GO, PEGylated-reduced GO | MTS, LDH, ROS | 50, 100, 150, 200, 300 µg/ml | HEK93T cells, MRC-5 cells | GO showed reduction on cell viability; PEG-rGO showed cytotoxicity on the highest concentration, but overall better viability then GO | Demirel et al. (2) |
| GO | Alamar blue assay, trypan blue, propidium iodide | 10, 50, 100 µg/ml | A549 cells | Dose- and time-dependent cytotoxicity | Mbeh et al. (3) |

| | | | | | |
|---|---|---|---|---|---|
| Polylactic acid graphene nanoplatelets | MTT, live/dead viability kit | 1, 5, 10 μg/ml | 3T3 cells | Dose- and time-dependent cytotoxicity | Pinto et al. (4) |
| rGO microfibers | live/dead viability kit, cell count kit-8 | non-significant | Neural stem cells | Biocompatibility of rGO microfibres; positive effects on cell attachment | Guo et al. (5) |

**Table S2.** In vivo toxicity of the graphene-based nanomaterials.

| Tested nanomaterial | Exposure | Concentration | animal | Findings | Reference |
|---|---|---|---|---|---|
| Graphene | Intratracheal administration | 0,12; 0,47; 1,88 $mg/m^3$; 6h/day | Male SD rats | BALF inflammatory parameters, no genotoxicity, no dose-dependent effects on body weight | Kim et al. (6) |
| Graphene oxide nanoplatelets (GON) | Intraperitoneal injection | 50, 150, 500 mg/kg; every 48 hours | Wistar rats | Alveolar thickening, small foci of an inflammatory reaction, hyperemia, hemosiderosis, giant cell formation, accumulation of GON in capsule, neuronal degeneration | Sharifabadi et al. (7) |
| PEG-nano GO | Peroral administration, intraperitoneal injection | 100 mg/kg | BALB/c mice | No effect on survival, body weight, morphology of liver, spleen, kidney, heart, lung | Yang et al. (8) |
| graphene oxide (GO), reduced GO,graphene quantum dots (GQD), aminated GQD, carboxyl GQD, graphene oxide GQD | Intravenous injection | 10 mg/kg | Kunming mice | GO, rGO caused weight loss, increase of white blood cells (inflammatory response), decrease of blood platelets; lungs contain GO and rGO aggregates (occlusion of blood vessels), and alveolar septa thickened; hydropic degeneration of hepatocytes | Li et al. (1) |

| | | | | | |
|---|---|---|---|---|---|
| Graphene nanoplatelets | Intratracheal administration | 50 μg/animal | Mice | Inflammation, apoptosis, pulmonary toxicity (pleural lavage) | Schinwald et al. (9) |

**References:**


1. Li, J.; Zhang, X.; Jiang, J.; Wang, Y.; Jiang, H.; Zhang, J.; Nie, X.; Liu, B. Systematic assessment of the toxicity and potential mechanism of graphene derivatives in vitro and in vivo. *Toxicol. Sci.* **2019**, *167*, 269–281.
2. Demirel, E.; Karaca, E.; Durmaz, Y.Y. Effective PEGylation method to improve biokompatibility of graphene derivatives. *Eur. Polym. J.* **2020**, *124*, 109504.
3. Mbeh, D.A.; Akhavan, O.; Javanbakth, T.; Mahmoudi, M.; Yahia, L. Cytotoxicity of protein corona-graphene oxide nanoribbons on human epithelial cells. *Appl. Surf. Sci.* **2014**, *320*, 596–601.
4. Pinto, A.M.; Moreira, S.; Goncalves, I.C.; Gama, F.M.; Mendes, A.M.; Magalhaes, F.D. Biocompatibility of poly(lactic acid) with incorporated graphene-based materials. *Colloids Surf. B* **2013**, *104*, 229–238.
5. Go, W.; Qiu, J.; Liu, J.; Liu, H. Graphene microfiber as a scaffold for regulation of neural stem cells differentiation. *Sci. Rep.* **2017**, *7*, 5678. https://doi.org/10.1038/s41598-017-06051-z.
6. Kim, J.K.; Shin, J.H.; Lee, J.S.; Hwang, J.H.; Lee, J.H.; Baek, J.E.; Kim, T.G.; Kim, B.W.; Kim, J.S.; Lee, G.H. et al. 28-day inhalation toxicity of graphene nanoplatelets in Sprague-Dawley rats. *Nanotoxicology* **2016**, *10*, 891–901.
7. Sharifabadi, M.A.; Koohi, M.K.; Zayerzadeh, E.; Hablolvarid, M.H.; Hassan, J.; Seifalian, A.M. In vivo toxicological evaluation of graphene oxide nanoplatelets for clinical application. *Int. J. Nanomed*. **2018**, *13*, 4757–4769.
8. Yang, K.; Gong, H.; Shi, X.; Wan, J.; Zhang, Y.; Liu, Z. In vivo distribution and toxikology of functionalized nano-graphene oxide in mice after oral and intraperitoneal administration. *Biomaterials* **2013**, *34*, 2787–2795.
9. Schinwald, A.; Murphy, F.A.; Jones, A.; Macnee, W.; Donaldson, K. Graphene-based nanoplatelets: A new risk to the respiratory system as a consequence of their unusual aerodynamic properties. *ACS Nano* **2012**, *6*, 736–746.
